# Interactive impact of potassium and arbuscular mycorrhizal fungi on the root morphology and nutrient uptake of sweet potato (*Ipomoea batatas* L.)

**DOI:** 10.3389/fmicb.2022.1075957

**Published:** 2023-01-09

**Authors:** Jie Yuan, Kun Shi, Xiaoyue Zhou, Lei Wang, Cong Xu, Hui Zhang, Guopeng Zhu, Chengcheng Si, Jidong Wang, Yongchun Zhang

**Affiliations:** ^1^Institute of Agricultural Resources and Environment, Jiangsu Academy of Agricultural Sciences, Nanjing, Jiangsu, China; ^2^National Agricultural Experimental Station for Agricultural Envrionment, Nanjing, Jiangsu, China; ^3^College of Horticulture, Hainan University, Haikou, Hainan, China

**Keywords:** sweet potato, arbuscular mycorrhizal fungi, potassium, nutrient uptake, root morphology, tuberous root yield

## Abstract

Sweet potato is a typical “potassium (K)-favoring” food crop and strongly dependent on arbuscular mycorrhizal fungi (AMF). Recent studies show the importance of K and AMF to morphology optimization and nutrient uptake regulation of sweet potato; meanwhile, the interaction exists between K and K use efficiency (KIUE) in sweet potato. To date, only a few studies have shown that AMF can improve plant K nutrition, and whether the benefits conferred by AMF on plant are related to K remains unclear. In this study, low-KIUE genotype “N1” and high-KIUE genotype “Xu28” were used as experimental sweet potato; *Funneliformis mosseae* (FM) and *Claroideoglomus etunicatum* (CE) were used as experimental AMF. In a pot experiment, plants “N1” and “Xu28” were inoculated with FM or CE, and applied with or without K fertilizer to uncover the effects of K application and AMF inoculation on the root morphology and nutrient absorption of sweet potato during their growing period. Results demonstrated that AMF inoculation-improved root morphology of sweet potato highly relied on K application. With K application, AMF inoculation significantly increased root tip number of “N1” in the swelling stage and optimized multiple root morphological indexes (total root length, root surface area, root volume, root diameter, root branch number, and root tip number) of “Xu28” and CE had the best optimization effect on the root morphology of “Xu28”. In addition, CE inoculation significantly promoted root dry matter accumulation of “Xu28” in the swelling and harvesting stages, coordinated aerial part and root growth of “Xu28”, reduced the dry matter to leaf and petiole, and was beneficial to dry matter allocation to the root under conditions of K supply. Another promising finding was that CE inoculation could limit K allocation to the aboveground and promote root K accumulation of “Xu28” under the condition with K application. The above results lead to the conclusion that K and CE displayed a synergistic effect on root development and K acquisition of high-KIUE “Xu28”. This study could provide a theoretical basis for more scientific application of AMF in sweet potato cultivation and will help further clarify the outcomes of plant-K–AMF interactions.

## Introduction

Many tuber and storage root crops owing to their high nutritional values offer high potential to overcome food security issues (Kondhare et al., 2021). Sweet potato [*Ipomoea batatas* (L.) Lam.], belongs to the Convolvulaceae family, is widely grown in tropical and subtropical climates worldwide (Minemba et al., 2019), and it is an essential root crop and a high-yield food, which guarantees food security and improves the nutritional status of people (Liu et al., 2019; Wang et al., 2022a). It is worth noting that the root system of sweet potato is involved not only in uptake of water and mineral nutrient but also in storage of photosynthate, and hence root growth and differentiation are closely related to tuberous root yield (Gao et al., 2021). Less root differentiation and low nutrient uptake efficiency are currently the main obstacles limiting the further improvement of sweet potato yield production. As documented, the average root diameter and root volume of sweet potato reflect the root differentiation, and the total root length, root surface area, and root tip number determine the water and nutrient absorption efficiency (Liu et al., 2017). Therefore, it is imperative to seek effective methods for root morphology and nutrient uptake optimization. Previous studies have reported that soil and rhizosphere microbial activities are the major factors that determine the availability of nutrients to plants and consequently have significant influences on root growth and plant productivity (Wang et al., 2017). Recent evidence suggests that fertilization optimization and microbial inoculation were effective tools for sweet potato cultivation (Minemba et al., 2019; Alhadidi et al., 2021).

Sweet potato is a typical versatile crop with higher requirements for K for optimum production throughout the growth period than cereals, oilseeds, and commercial crops (Tang et al., 2015). Over the past few years, studies have proven that K was quite important for root system development and the final tuberous root yield of sweet potato (Gao et al., 2021). As estimated, about 10 kg K was required to produce 1,000 kg (on dry matter [DM] basis) tuberous root of sweet potato (George et al., 2002). The K utilization efficiency (KIUE) was interlinked with K uptake (Wang et al., 2017). Using K-efficient genotypes in combination with appropriate soil fertilization worked as an optimal K nutrient management strategy for sustainable and environmentally protective crop production (Romheld and Kirkby, 2010). In 1993, a study reported the significant interaction between environment and K supply on sweet potato biomass production and KIUE (Woodend and Glass, 1993). Our previous studies have selected 5 out of 108 sweet potato genotypes based on the significant differences in KIUE among the field experiment (Wang et al., 2015). Then, we investigated the interactions between environmental factors and KIUE genotype of sweet potato and found that high-KIUE genotype had a more optimal partitioning of K to economic sink as well as higher K was associated with the resource–sink relationship at different growth stages than did low-KIUE genotype (Wang et al., 2017).

Arbuscular mycorrhizal fungi (AMF) could live in a mutualistic symbiosis with plant roots of the majority (> 80%) of terrestrial plant species (Kalamulla et al., 2022; Seemakram et al., 2022). Sweet potato was easily colonized by mycorrhizal fungi (O’Keefe and Sylvia, 1993; Minemba et al., 2019). AMF varied in their ability to establish after inoculation, and in their effect on the yield and quality of sweet potato tubers (Farmer et al., 2007). Recently, some beneficial effects of AMF on the performance of sweet potato were reported (Tong et al., 2013; Yooyongwech et al., 2016; Alhadidi et al., 2021), such as water-deficit tolerance improvement, β-carotene concentration increase, root morphology regulation, and nutrient acquisition alternation. Therefore, the beneficial interaction of AMF with sweet potato is considered suitable for the cultivation of sweet potato. Typically, AMF develop extraradical mycelia that extend the depletion zone that develops around roots, and facilitate the acquisition of nutrients of low mobility (Seemakram et al., 2022). Consistent with this phenomenon, a meta-analysis about the effects of AMF on plant growth and nutrient uptake reflected that AMF could increase plant nitrogen (N), phosphorus (P), and K uptake by 22.1, 36.3, and 18.5%, respectively (Chandrasekaran, 2020). It is important to note that the enhanced nutrient uptake by AMF is ascribed to regulation in root elongation, lateral root and root hair formation, and root surface area and root volume expansion (Chandrasekaran, 2020). However, only a few studies have shown that AMF can improve plant K nutrition (Garcia and Zimmermann, 2014). To date, there is still no report on the effects of AMF inoculation on K uptake of sweet potato, and whether the benefits conferred by AMF on plants are related to K remains unknown. Given the importance of K and AMF to morphology optimization and nutrient uptake regulation of sweet potato (Minemba et al., 2019; Alhadidi et al., 2021), the interaction exists between K and KIUE in sweet potato (Wang et al., 2017); hence, a global understanding of the interactive impact of K and AMF on the tuberous root yield, root morphology, and nutrient uptake of different KIUE genotypes of sweet potato should be considered.

In this study, low KIUE genotype “N1” and high KIUE genotype “Xu28” were used as the experimental varieties of sweet potato based on our previous results (Wang et al., 2015, 2017); *Funneliformis mosseae* (FM) and *Claroideoglomus etunicatum* (CE) were used as the experimental AMF mainly considering the existing studies (Gai et al., 2006; Alhadidi et al., 2021). In a pot experiment, the plants “N1” and “Xu28” were inoculated with FM or CE, and applied with or without K fertilizer to uncover the effects of K application and AMF inoculation on the root morphology and nutrient absorption of sweet potato during their growing period. The following three essential questions were addressed throughout this article: (1) How K application and AMF inoculation differentially affect root morphology and nutrient uptake of low-KIUE and high-KIUE sweet potato? (2) Whether there are interactive effects on the root morphology and nutrient uptake between K application and AMF inoculation? and (3) Which is the best combination of K (with or without K application) and AMF (FM or CE inoculation)?

## Materials and methods

### Soil, plant, and AMF materials

The soil was sandy loam fluvo-aqic (Eutyic Cambisol) collected from Jiangyan City, Jiangsu Province (Wang et al., 2017). Before the experiment began, plant debris and rocks were removed from the soil. The soil sample used in this study contained (on a DM basis) 52.01 g kg^–1^ alkali-hydrolyzable N; 12.15 mg kg^–1^ Olsen-P; 53 mg kg^–1^ available K (with NH_4_OAC extraction); and 15.1 mg kg^–1^ organic matter. The pH of the soil was 7.13 in a 1:2.5 (w/v) soil–water suspension ratio.

The two sweet potato genotypes “N1” and “Xu28” were selected based on K uptake and K-use efficiency in the previous studies (Wang et al., 2015, 2017, 2018), with “N1” bred by the Institute of Food Crops, Jiangsu Academy of Agricultural Sciences, exhibiting low K uptake and K-use efficiency, while “Xu28” bred by the Jiangsu Xuzhou Sweet Potato Research Central, exhibiting high K uptake and K-use efficiency.

The original AMF inoculum FM (BGC XJ02) and CE (BGC XJ03C) were purchased from the Bank of Glomales in China (BGC), Institute of Plant Nutrition and Resources, Beijing Academy of Agriculture and Forestry Sciences (Beijing, China). AMF spores were germinated with *Trifolium repens* as host plant in sterilized fine sand and vermiculite culture in a greenhouse of the Jiangsu Academy of Agricultural Sciences (Nanjing, China, 119°32′21″E, 31°44′ 03″N) over 3 months (Xu et al., 2021). The AMF inoculum for both FM and CE consisted of spores (∼42 spores g^–1^), external hyphae, infected root in addition to fine sand and vermiculite.

### Experimental procedure

A greenhouse experiment was conducted in the greenhouse of the Jiangsu Academy of Agricultural Sciences (Nanjing, China, 119°32’21″E, 31°44’ 03″N) over 4 months from August to November 2021. Two different K treatment (0 or 160 mg K_2_SO_4_ per kg air-dried soil) were applied in the presence or absence of AMF (FM or CE) for two sweet potato genotypes (“N1” or “Xu28”). Each plastic pot (height = 30 cm, diameter = 30 cm) was filled with 7 kg air-dried soil; the soil contained 0.82 g urea, 1.55 g superphosphate, and either without K_2_SO_4_ (K0) or with 1.12 g K_2_SO_4_ (K1). In total, 16 (cultivar × K treatment × AMF inoculation) treatments include (1) N1/−K/−FM, (2) N1/−K/+FM, (3) N1/−K/−CE, (4) N1/−K/+CE, (5) N1/+K/−FM, (6) N1/+K/+FM, (7) N1/+K/−CE, (8) N1/+K/+CE, (9) Xu28/−K/−FM, (10) Xu28/−K/+FM, (11) Xu28/−K/−CE, (12) Xu28/−K/+CE, (13) Xu28/+K/−FM, (14) Xu28/+K/+FM, (15) Xu28/+K/−CE, and (16) Xu28/+K/+CE. For +FM or +CE, the AMF inoculum (70 g) of FM or CE was inoculated into each pot at the depth of 10 cm, respectively. For −FM or −CE, the same amount of AMF inoculum (70 g) of FM or CE was sterilized three times (121°C, 20 min) and filtered through a 25-μm membrane, respectively. The obtained microbial filtrate (50 ml) of FM or CE inoculum was inoculated into the pot of −FM or −CE, respectively. Each pot contained one plant, and there were 12 pots of each of the 16 (genotype × K treatment × AMF inoculation) combinations.

### Plant sampling and analysis

Three pots for 16 treatments were randomly selected at plantlet stage [S1, 30 days post inoculation (DPI)], swelling stage (S2, 60 DPI), maturing stage (S3, 85 DPI), and harvesting stage (S4, 120 DPI). Three sweet potato plants from three individual pots were destructively sampled and regarded as three independent biological replicates.

Plant samples were separated into leaves, stems, stalks, and roots for fresh matter (FM) measurement, and fresh root samples were used for the evaluation of root morphological characteristics and the analysis of mycorrhizal colonization. Additionally, the root parts were divided into fibrous roots and tuberous roots at harvesting stage. Then, leaves, stem, petiole, and root were oven-dried at 105°C for 1 h and then at 75°C to constant weight for DM measurement, then ground and passed through a 0.5-mm sieve for K concentration analysis.

Root morphology of sweet potato was characterized using an Epson Perfection V850 Pro scanner (Epson, Nagano, Japan) at 300 dpi. Root morphological characteristics, including the root length (RL), total root length (RL), root surface area (RA), average root diameter (RD), root volume (RV), root tip number (RT), root branch number (RB), and root cross number (RC), were estimated using WinRHIZO Pro 2017a Root Analysis System (Regent Instruments Inc., Canada) (Huang et al., 2019).

Mycorrhizal root colonization intensity was assessed. The fresh fine roots (0.5 g) were cleared with 10% KOH for 60 min at 90°C, acidified in 2% HCl for 30 min, and then stained with ink (Sheaffer Pen, Shelton, CT, USA) and the vinegar method at 90°C for 15 min. Then, about 90 root segments were randomly selected from each sample and mounted on the microscope slides. Mycorrhizal colonization of root was determined using a microscope at 40× magnification (SMZ745T Nikon, Japan) (Phillips and Hayman, 1970).

Dried plant samples passed through 0.5-mm sieve (0.5 g) were digested with a mixture of H_2_SO_4_ and H_2_O_2_, then the K concentration was determined by flame photometry (Wang et al., 2017). The plant K uptake was calculated as follows:


(1) K⁢uptake=DM×K⁢concentration

### Statistical analysis

Means and standard errors (SE) were calculated using SPSS Statistics 20.0 software (SPSS Inc., Chicago, IL, USA) (Wang et al., 2017; Yuan et al., 2019). Values are the means of three independent experiments. Bars represent standard errors. Before the analysis of variance (ANOVA), the normality and homogeneity of variance were checked. For the normality test of variance, Shapiro–Wilk test was conducted when the date is less than 50. When the *P* value is >0.05, the data have the characteristic of normal distribution. If the absolute value of kurtosis is <10 and the absolute value of skewness is <3, the data can be accepted as normal distribution basically. The normal distribution histogram and regression analysis was observed intuitively to check whether this set of data roughly conforms to the normal distribution. For the homogeneity test of variance, when the *P* value is >0.05, the data conforms to homogeneity of variance. The statistical evaluation between two treatments was compared using the independent-samples *t*-test, and asterisks denote significant differences between two treatments (*t*-test; *P < 0.05; **P < 0.01; ***P < 0.001). The statistical evaluation of more than two treatments was compared using one-way ANOVA, followed by Tukey’s multiple-comparison test (*P* < 0.05), and different lowercase letters denote significant differences. Two-way ANOVA was used to test the statistical significance of K application (K), FM inoculation (FM), CE inoculation (CE), K application × FM inoculation interaction (K × FM), and K application × CE inoculation interaction (K × CE) using the general linear model (GLM). Three-way ANOVA was used to test the statistical significance of Genotype (G), K application (K), AMF inoculation (AMF), Genotype × K application interaction (G × K), Genotype × AMF inoculation interaction (G × AMF), K application × AMF inoculation interaction (K × AMF), and Genotype × K application × AMF inoculation interaction (G × K × AMF) using the GLM. Pearson’s correlation relationship was analyzed using Origin 2021 and displayed as heatmap format.

## Results

### AMF colonized in the root of sweet potato

**FIGURE 1:**
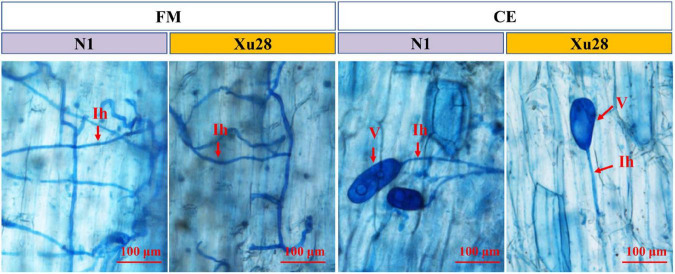
Microscopic visualization of arbuscular mycorrhizal fungi structures in root of sweet potato at seeding stage. “N1” and “Xu28” indicate “sweet potato “N1”” and “sweet potato “Xu28””, respectively. “FM” and “CE” indicate “*Funneliformis mosseae*” and “*Claroideoglomus etunicatum*”, respectively. Red arrows indicate different arbuscular mycorrhizal fungi (AMF) structures. Ih, intraradical hyphae; V, vesicle.

*Funneliformis mosseae* and *Claroideoglomus etunicatum* could establish symbiotic relationship with sweet potato for both “N1” and “Xu28” (Figure 1 and Supplementary Figure 1). ANOVA revealed that genotype significantly affects the colonization rate of FM and CE at harvesting stage and seeding stage, respectively. K application significantly affects AMF colonization rate in root of “N1” at harvesting stage, and K × AMF significantly affects AMF colonization rate in root of “Xu28” at swelling stage. No significant differences were observed in FM and CE colonization rate in the root of “N1” and “Xu28” from seeding stage to harvesting stage, except that FM exhibited significant higher colonization rate in the root of “N1” than that of “Xu28” at the harvesting stage (Supplementary Figure 1). K application had no significant effects on FM or CE colonization rate in the root of “N1” or “Xu28”, except that K application significantly increased FM colonization rate in the root of “N1” at the harvesting stage. Therefore, FM preferred to colonize in the root of “N1” rather than of “Xu28” at the harvesting stage, and K application contributed to colonization of FM in the root of “N1” at the harvesting stage.

### K and AMF improved the tuberous root yield of sweet potato

**FIGURE 2:**
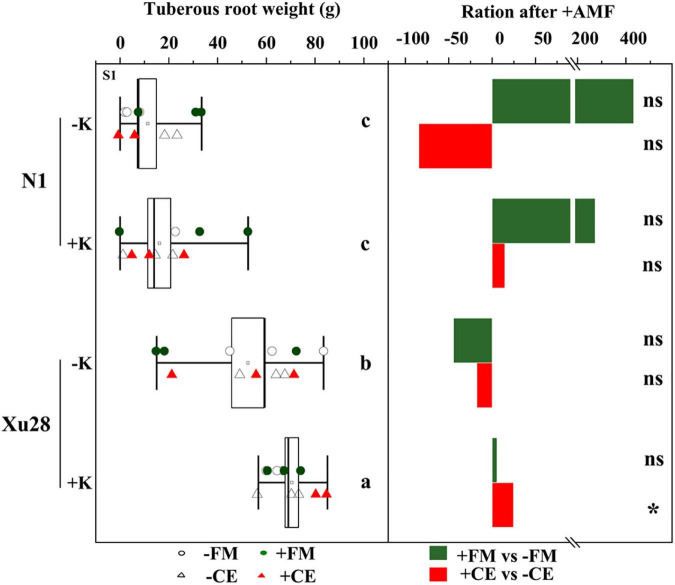
The effects of arbuscular mycorrhizal fungi inoculation on tuberous root yield of sweet potato. Different lowercase letters indicate significant differences between different groups (*P* < 0.05). Asterisks denote significant differences after arbuscular mycorrhizal fungi (AMF) inoculation (*t*-test; *P < 0.05), while “ns” indicates nonsignificant difference.

Potassium application had no significant effects on the tuberous root yield of “N1”, while K application significantly increased the tuberous root yield of “Xu28” (Figure 2). No significant differences were observed in the tuberous root yield of “N1” after FM or CE inoculation, and no significant differences were observed in the tuberous root yield of “Xu28” after FM inoculation, while CE inoculation significantly increased the tuberous root yield of “Xu28” under K application condition. Therefore, FM or CE inoculation did not have a significant effect on improving the tuberous root yield of “N1”, while CE inoculation could effectively improve the tuberous root yield of “Xu28” under the condition with K application, and K application and CE inoculation exhibited a synergistic effect on the tuberous root yield of “Xu28”. K application and CE inoculation combination increased tuberous root yield of “Xu28” by 37.66% compared with the condition without K application and uninoculated with CE.

### K and AMF optimized root morphology of sweet potato

**FIGURE 3:**
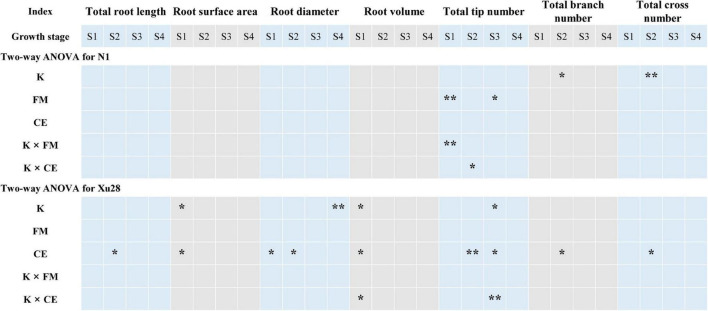
Two-way ANOVA for the effects potassium (K) and *F. mosseae* (FM) or *C. etunicatum* (CE) inoculation on root morphological traits of sweet potato. Asterisks denote significant differences (*t*-test; *P < 0.05; **P < 0.01).

Analysis of variance revealed that K application significantly affects total root length and root branch number of “N1” in the swelling stage (Figure 3). For “Xu28”, K significantly affects root surface area and root volume in the seeding stage, root tip number in the swelling stage, and root diameter in the harvesting stage. Thus, K application differentially regulated the root architecture of “N1” and “Xu28”, and K application showed greater effects on root architecture optimization of “Xu28”. ANOVA showed that FM significantly regulated the root tip number of “N1” in the seeding and maturing stages, while CE had no significant effects on the root morphology of “N1”. For “Xu28”, FM had no significant effects on the root morphology, while CE significantly affects root surface area, root diameter, root volume in the seeding stage, and root total root length, root diameter, root tip number, root branch number and root cross number in the swelling stage, and root tip number in the maturing stage. Therefore, FM and CE inoculation were more inclined to optimize root morphology of “N1” and “Xu28”, respectively. In particular, FM inoculation only affected “N1” root tip number, while CE inoculation affected several indexes of “Xu28” root morphology.

**FIGURE 4:**
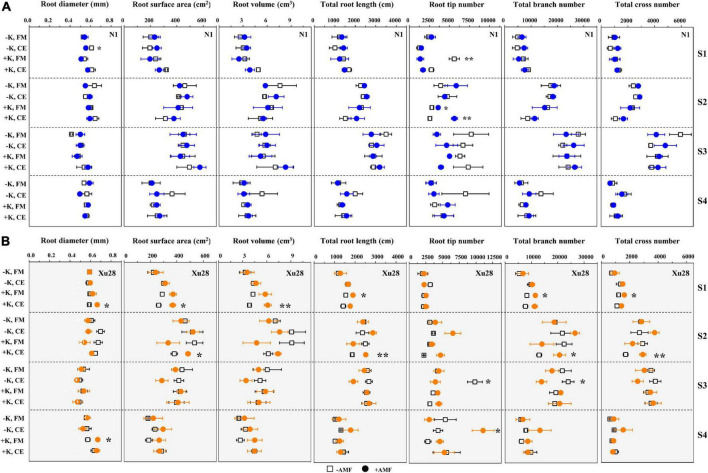
The effects of potassium application and arbuscular mycorrhizal fungi (AMF) inoculation on the root morphology of N1 **(A)** and Xu28 **(B)**. For different groups, asterisks denote significant differences after AMF inoculation (*t*-test; *P < 0.05; **P < 0.01).

As shown in Figure 4 and Supplementary Figure 2, FM and K × FM significantly affected root tip number at seeding stage of “N1”, and K, CE, and K × CE significantly affected root volume at seeding stage and root tip number at maturing stage of “Xu28”. Meanwhile, we analyze the effects of FM and CE inoculation on the root morphology of “N1” and “Xu28” without K application (Figure 4). For “N1”, FM inoculation had no significant effects on the root morphology, while CE inoculation significantly decreased root diameter in the seeding stage and root tip number in the maturing stage, and increased root tip number in the harvesting stage. For “Xu28”, FM inoculation had no significant effects on the root morphology, and CE inoculation significantly decreased root tip number in the maturing stage. Subsequently, we further analyze the effects of FM and CE inoculation on the root morphology of “N1” and “Xu28” with K application (Figure 4). For “N1”, FM inoculation significantly decreased root tip number in the seeding stage and increased root tip number in the swelling stage, CE inoculation significantly increased root tip number in the swelling stage. For “Xu28”, FM inoculation significantly increased total root length in the seeding stage and root diameter in the harvesting stage, CE inoculation significantly increased root surface area, root volume, root diameter, and root branch number in the seeding stage, and increased total root length, root surface area, root tip number, and root branch number in the swelling stage. As a result, FM or CE inoculation had little effect on the root morphology of sweet potato and tended to have a negative effect without K application, while FM and CE inoculation significantly increased root tip number of “N1” in the swelling stage and optimized multiple indexes of root morphology of “Xu28” during the whole growth stage under the condition with K application, indicating that K application and AMF inoculation exhibited a considerable synergistic effect on the root morphology of “Xu28”.

In addition, CE had the best optimization effect on the root morphology of “Xu28” among the whole growth period under the condition with K application. The longer the total root length and the larger the root surface area, the more effective the plant roots to absorb water and nutrients. The more the root branch number, the more the root tip number. Root tip was the main organ of root nutrient absorption, and root branch number was positively correlated with nutrient absorption capacity. Accordingly, CE inoculation under the condition with K application has the potential to promote root nutrient absorption of “Xu28” at seedling and swelling stages.

### K and AMF regulated plant growth parameters of sweet potato

Potassium application had no significant effect on total DM of “N1” from seeding stage to harvesting stage (Supplementary Figure 3). For “Xu28”, K application had no significant effect on total DM from swelling stage to harvesting stage, while significantly increased total DM at the seeding stage. In addition, no significant differences were observed in total DM of “N1” or “Xu28” after FM or CE inoculation. ANOVA revealed that FM, CE, K × FM, and K × CE had no significant effect on total DM of “N1” or “Xu28” (Supplementary Figure 4). Thus, K application favored total DM accumulation of “Xu28” at the seeding stage, while FM and CE had no inspiration effect on total DM of “N1” and “Xu28” with or without K application.

**FIGURE 5:**
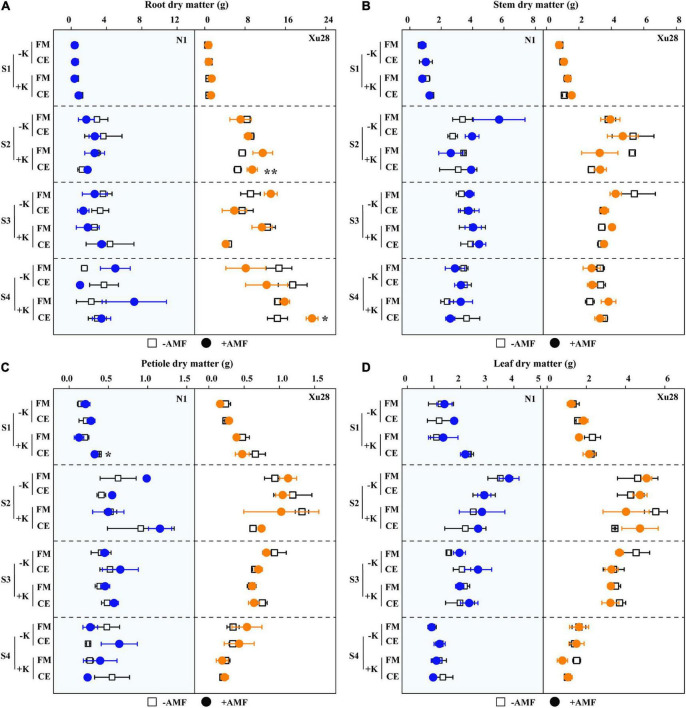
The effects of potassium application and arbuscular mycorrhizal fungi (AMF) inoculation on dry matter accumulation of root **(A)**, stem **(B)**, petiole **(C)**, and leaf **(D)** of sweet potato. For different groups, asterisks denote significant differences after AMF inoculation (*t*-test; *P < 0.05; **P < 0.01).

Dry matter accumulation of different organs of sweet potato were analyzed. ANOVA revealed that K, FM, CE, K × FM, and K × CE had no significant effect on root, stem, petiole, or leaf DM of “N1” from seeding stage to harvesting stage, except that K significantly affected leaf DM at swelling stage (Supplementary Figure 4). For “Xu28”, K, CE, and K × CE significantly affected root DM of “Xu28” at seeding stage, and K significantly affected stem, petiole, leaf DM of “Xu28” at seeding and harvesting stage (Supplementary Figure 4). As shown in Figure 5, no significant differences were observed in root, stem, petiole, or leaf DM of “N1” or “Xu28” after FM inoculation with or without K application, and no significant differences were observed in root, stem, petiole, or leaf DM of “N1” or “Xu28” after CE inoculation without K application. With K application, CE significantly decreased petiole DM of “N1” in the seeding stage, and significantly promoted root DM accumulation of “Xu28” in the swelling and harvesting stages (Figure 5). As a result, K application and CE inoculation exerted a synergistic effect on root DM of “Xu28” at seeding stage.

**FIGURE 6:**
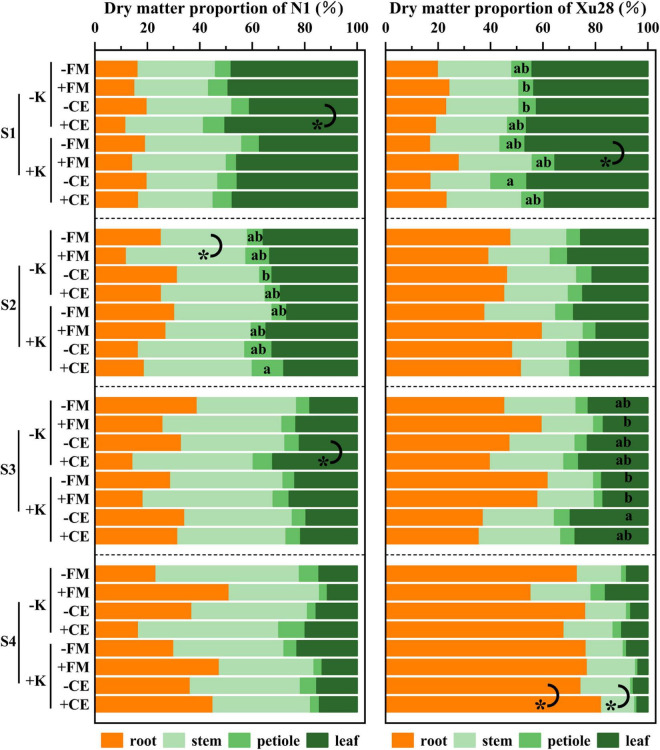
The effects of potassium application and arbuscular mycorrhizal fungi inoculation on dry matter proportion of different organs of sweet potato. For each growth stage, different lowercase letters indicate significant differences between different groups (*P* < 0.05). For different groups, asterisks denote significant differences after arbuscular mycorrhizal fungi (AMF) inoculation (*t*-test; *P < 0.05).

Furthermore, we further analyze DM proportion of different organs. For “N1”, CE inoculation resulted in significantly higher DM proportion of leaf in the seeding and maturing stages without K application, FM inoculation resulted in significantly higher DM proportion of stem in the swelling stage without K application (Figure 6). For “Xu28”, FM inoculation resulted in significantly lower DM proportion of leaf in the seeding stage with K application, CE inoculation resulted in significantly higher DM proportion of root and lower DM proportion of stem in the harvesting stage with K application (Figure 6). ANOVA revealed that K × CE significantly affected root and aboveground DM proportion (Supplementary Figure 5). The above results indicated that CE inoculation under the condition of K application significantly coordinated the aerial part and root growth of “Xu28” at seeding stage, reduced the DM to leaf and petiole at harvesting stage, and be beneficial to DM allocation to the root at seeding and swelling stages.

### K and AMF coordinated plant K uptake and allocation

**FIGURE 7:**
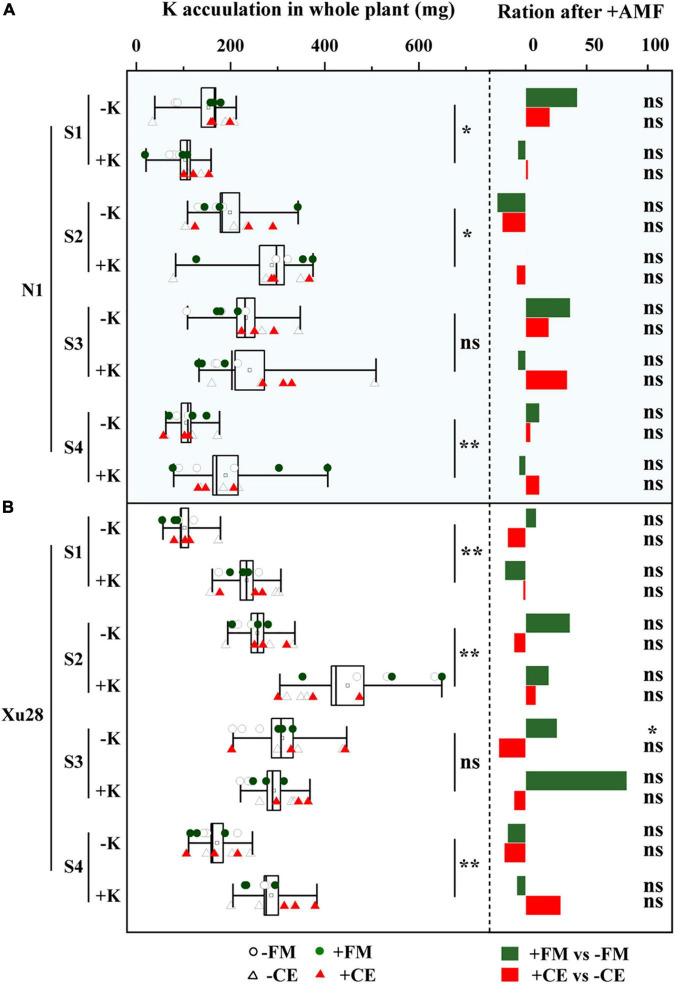
The effects of potassium application and arbuscular mycorrhizal fungi inoculation on K concentration **(A)** and K accumulation **(B)** of sweet potato. For each growth stage, different lowercase letters indicate significant differences between different groups (*P* < 0.05). For different groups, asterisks denote significant differences after arbuscular mycorrhizal fungi (AMF) inoculation (*t*-test; *P < 0.05; **P < 0.01), while “ns” indicates nonsignificant difference.

As shown in Figure 7, K application significantly increased K concentration and K accumulation of “N1” and “Xu28” at seeding stage, swelling stage, and harvesting stage. No significant differences were observed in K concentration and K accumulation of “N1” or “Xu28” after AMF inoculation with or without K application, except that FM significantly reduced K concentration at maturing stage of “Xu28” under the condition with K application and FM significantly increased K accumulation at maturing stage of “Xu28” under the condition without K application.

Potassium accumulation in different organs of sweet potato was analyzed (Table 1). For “N1”, FM inoculation significantly increased petiole K accumulation at swelling and maturing stages and leaf K accumulation at maturing stage under the condition without K application, while significantly decreased leaf K accumulation at maturing stage under the condition with K application. While CE inoculation had no significant effect on K accumulation in different organs of “N1” under the condition with or without K application. Thus, compared with K application, FM inoculation was more conducive to aerial part K accumulation of “N1” under the condition without K application. For “Xu28”, FM and CE had no significant effect on K accumulation in different organs under the condition without K application, while FM inoculation significantly increased leaf K accumulation at maturing stage and CE inoculation significantly increased root K accumulation at seeding stage under the condition with K application, showing that CE-increased root K accumulation at seeding stage and FM-increased leaf K accumulation at maturing stage of “Xu28” were both dependent on K application.

**TABLE 1:** The effects of K application and arbuscular mycorrhizal fungi (AMF) inoculation on K accumulation in different organs of sweet potato.

G	K	AMF	Root K accumulation (mg)				Stem K accumulation (mg)				Petiole K accumulation (mg)				Leaf K accumulation (mg)			
			S1	S2	S3	S4	S1	S2	S3	S4	S1	S2	S3	S4	S1	S2	S3	S4
N1	−K	−FM	10.03a	47.68a	60.52a	16.99a	47.57a	39.70a	61.17b	40.17a	12.02a	16.52a	14.23b	13.77a	48.49a	59.13a	38.84a	19.87a
		+FM	11.11a	28.39a	44.23a	57.52a	50.05a	81.76a	68.26ab	31.38a	19.58a	33.90a**	23.66ab*	6.75a	87.25a	78.58a	53.68a*	18.53a
		−CE	14.66a	56.21a	73.12a	39.51a	53.79a	49.06a	136.35a	46.20a	19.55a	16.67a	35.46ab	6.50a	60.07a	64.32a	59.93a	29.79a
		+CE	9.51a	45.54a	22.34a	11.58a	61.46a	67.99a	126.05ab	36.52a	26.27a	22.46a	43.66a	18.90a	80.22a	85.81a	67.68a	28.01a
	+K	−FM	5.80a	65.86a	47.01a	42.16a	34.48a	127.06a	65.70ab	58.67a	12.30a	34.68a	13.59b	10.76a	29.49a	78.19a	59.64a	32.18a
		+FM	5.92a	57.87a	24.91a	120.55a	29.68a	104.35a	65.86ab	91.64a	5.81a	30.05a	16.14ab	21.88a	35.32a	93.82a	47.35a*	28.97a
		−CE	16.49a	20.55a	95.19a	47.16a	36.24a	87.73a	132.06ab	84.62a	16.63a	52.13a	31.16ab	19.32a	57.86a	77.84a	55.77a	32.62a
		+CE	16.13a	35.78a	77.7a	61.18a	43.84a	129.33a	125.92ab	67.66a	17.27a	66.10a	36.79ab	11.54a	52.64a	88.43a	66.85a	25.69a
Xu28	−K	−FM	8.64B	114.99B	119.86A	125.78B	20.75B	22.16B	28.30B	11.22B	13.72B	11.53B	13.80B	7.27A	56.27A	74.34B	69.34A	26.49A
		+FM	8.70B	110.35B	210.00A	95.89B	15.63B	24.67B	26.66B	12.35B	7.61B	16.50B	17.88B	11.06A	44.21A	96.64AB	60.81A	25.98A
		−CE	11.26AB	132.37B	164.76A	153.03B	24.18B	34.39B	68.24A	13.91B	11.75B	20.18B	45.14A	8.96A	81.24A	85.68B	85.78A	26.55A
		+CE	9.94B	127.42B	108.35A	117.65B	20.82B	27.19B	78.87A	11.33B	14.57B	36.83AB	56.12A	8.61A	58.32A	92.04AB	85.15A	29.30A
	+K	−FM	16.77AB	154.81AB	282.72A	190.73AB	60.92A	98.10A	15.48B	33.24AB	41.77B	84.76A	9.11B	12.98A	102.59A	207.08A	46.03A	37.35A
		+FM	35.71A	243.49A	181.20A	181.81AB	59.01A	51.57AB	24.41B	47.16A	34.62B	66.41AB	14.11B	6.19A	92.72A	153.41AB	60.24A*	18.82A
		−CE	19.64AB	140.41B	117.80A	203.75AB	52.55A	47.82AB	65.11A	34.22AB	88.15A	34.68AB	49.59A	6.68A	95.64A	124.79AB	80.56A	25.19A
		+CE	28.75AB*	168.98AB	105.00A	295.62A	59.10A	45.51B	76.75A	27.13AB	40.54B	35.69AB	47.67A	5.23A	108.16A	136.61AB	109.83A	19.53A
N1	K		Ns	Ns	Ns	Ns	*	***	Ns	***	Ns	**	Ns	Ns	**	Ns	Ns	Ns
	FM		Ns	Ns	Ns	Ns	Ns	Ns	Ns	Ns	Ns	Ns	*	Ns	Ns	Ns	Ns	Ns
	CE		Ns	Ns	Ns	Ns	Ns	Ns	Ns	Ns	Ns	Ns	Ns	Ns	Ns	Ns	Ns	Ns
	K × FM		Ns	Ns	Ns	Ns	Ns	Ns	Ns	Ns	Ns	Ns	Ns	Ns	Ns	Ns	**	Ns
	K × CE		Ns	Ns	Ns	Ns	Ns	Ns	Ns	Ns	Ns	Ns	Ns	Ns	Ns	Ns	Ns	Ns
Xu28	K		***	**	Ns	***	***	***	Ns	***	***	**	Ns	Ns	**	**	Ns	Ns
	FM		Ns	Ns	Ns	Ns	Ns	Ns	Ns	Ns	Ns	Ns	*	Ns	Ns	Ns	Ns	Ns
	CE		Ns	Ns	Ns	Ns	Ns	Ns	Ns	Ns	Ns	Ns	Ns	Ns	Ns	Ns	Ns	Ns
	K × FM		Ns	Ns	Ns	Ns	Ns	Ns	Ns	Ns	Ns	Ns	Ns	Ns	Ns	Ns	Ns	Ns
	K × CE		*	Ns	Ns	Ns	Ns	Ns	Ns	Ns	Ns	Ns	Ns	Ns	Ns	Ns	Ns	Ns

Different lowercase letters in the same column indicate significant differences between different groups (*P* < 0.05) for “N1” or “Xu28”. Asterisks denote significant differences after AMF inoculation (*t*-test; *P < 0.05; **P < 0.01; ***P < 0.001). Ns denotes not significant using Tukey’s test.

Furthermore, we analyzed the proportion of K accumulation in different organs (Table 2). Either FM or CE inoculation had no significant effect on K proportion in different organs of “N1” and “Xu28” under the condition without K application. With K application, FM inoculation significantly decreased petiole K proportion at seeding stage of “N1” and stem K proportion at swelling of “Xu28”. ANOVA revealed that K, CE, and K × CE significantly affected petiole K proportion at seeding stage of “Xu28”, and the opposite effects on petiole K proportion of CE inoculation under the condition with and without K application, and CE inoculation significantly decreased petiole K proportion, showing that CE-decreased petiole K proportion of “Xu28” at seeding stage was highly dependent on K application. In addition, with K application, CE inoculation significantly decreased K proportion in leaf and stem at harvesting stage, and meanwhile significantly increased root K proportion at harvesting stage, indicating that K application and CE inoculation displayed a synergistic effect on K allocation in different organs of “Xu28”, and in particular: CE inoculation could limit K allocation to the arial part and be beneficial to K allocation to the root of “Xu28” at harvesting stage under the condition with K application.

**TABLE 2:** The effects of K application and arbuscular mycorrhizal fungi (AMF) inoculation on the proportion of K accumulation in different organs of sweet potato.

G	K	AMF	Root K proportion (%)				Stem K proportion (%)				Petiole K proportion (%)				Leaf K proportion (%)			
			S1	S2	S3	S4	S1	S2	S3	S4	S1	S2	S3	S4	S1	S2	S3	S4
N1	−K	−FM	8.59a	27.25a	31.75a	18.58a	41.71a	25.06a	35.42a	44.53a	10.02a	10.39a	9.23a	15.44a	39.67a	37.30a	23.60ab	21.44a
		+FM	6.66a	10.28a	21.83a	46.99a	29.49a	35.11a	36.89a	29.67a	11.61a	17.53a	12.52a	6.02a	52.24a	37.08a	28.76ab	17.32a
		−CE	12.40a	26.15a	23.94a	28.05a	33.19a	29.24a	45.18a	39.74a	11.85a	9.72a	11.42a	6.07a	42.57a	34.88a	19.46ab	26.14a
		+CE	5.24a	20.11a	8.12a	12.03a	34.67a	30.30a	49.53a	39.95a	14.97a	11.27a	16.32a	19.27a	45.12a	38.32a	26.03ab	28.75a
	+K	−FM	7.32a	21.41a	25.09a	22.39a	42.26a	41.65a	35.15a	42.14a	14.81a	11.29a	7.47a	8.17a	35.61a	25.65a	32.29a	27.31a
		+FM	6.26a	17.04a	13.83a	37.07a	43.70a	37.85a	43.70a	41.26a	5.99a*	10.26a	10.97a	6.88a	44.05a	34.85a	31.50ab	14.79a
		−CE	13.15a	7.63a	25.25a	28.80a	28.09a	36.57a	44.96a	44.57a	13.05a	18.76a	11.17a	9.65a	45.71a	37.03a	18.61b	16.99a
		+CE	12.83a	10.96a	25.11a	35.16a	33.69a	40.70a	41.10a	41.95a	13.36a	20.66a	12.22a	6.82a	40.12a	27.68a	21.58ab	16.07a
Xu28	−K	−FM	8.99A	52.21A	50.18ABC	73.43A	21.41A	9.87A	12.74AB	6.66A	13.52B	5.06A	6.15BC	4.41A	56.09A	32.86A	30.94A	15.50A
		+FM	10.86A	43.50A	66.45AB	60.92A	20.30A	10.21A	8.53B	9.72A	10.13B	6.75A	5.70BC	8.82A	58.70A	39.54A	19.31A	20.53A
		−CE	9.09A	49.35A	43.75ABC	73.22A	19.47A	12.64A	19.43AB	7.47A	9.54B	7.34A	12.94ABC	4.96A	61.89A	30.66A	23.88A	14.35A
		+CE	9.43A	45.79A	30.59C	66.58A	20.08A	9.49A	27.02A	7.42A	13.91B	12.45A	19.57A	6.53A	56.57A	32.27A	22.81A	19.46A
	+K	−FM	7.67A	28.80A	74.64A	69.48A	27.85A	17.80A	5.91B	12.15A	18.41B	15.58A	3.17C	4.74A	46.07AB	37.82A	16.27A	13.63A
		+FM	15.63A	50.76A	64.47AB	72.01A	26.64A	9.32A*	8.74B	18.28A	15.54B	11.60A	5.15BC	2.44A	42.19AB	28.33A	21.64A	7.27A
		−CE	8.27A	40.40A	37.59BC	75.70A	22.97A	13.69A	21.00AB	12.67A	34.62A	9.95A	16.03AB	2.41A	34.14B	35.97A	25.37A	9.22A
		+CE	12.59A	44.02A	31.13C	85.03A*	24.79A	11.80A	22.50AB	7.90A*	16.86B*	9.46A	13.98ABC	1.48A	45.76AB	34.73A	32.39A	5.58A*
N1	K		Ns	Ns	Ns	Ns	Ns	**	Ns	Ns	Ns	Ns	Ns	Ns	Ns	Ns	Ns	Ns
	FM		Ns	Ns	Ns	Ns	Ns	Ns	Ns	Ns	Ns	Ns	Ns	Ns	*	Ns	Ns	Ns
	CE		Ns	Ns	Ns	Ns	Ns	Ns	Ns	Ns	Ns	Ns	Ns	Ns	Ns	Ns	*	Ns
	K × FM		Ns	Ns	Ns	Ns	Ns	Ns	Ns	Ns	*	Ns	Ns	Ns	Ns	Ns	Ns	Ns
	K × CE		Ns	Ns	Ns	Ns	Ns	Ns	Ns	Ns	Ns	Ns	Ns	Ns	Ns	Ns	Ns	Ns
Xu28	K		Ns	Ns	Ns	Ns	*	Ns	Ns	*	**	*	Ns	*	***	Ns	Ns	**
	FM		Ns	Ns	Ns	Ns	Ns	*	Ns	Ns	Ns	Ns	Ns	Ns	Ns	Ns	Ns	Ns
	CE		Ns	Ns	Ns	Ns	Ns	Ns	Ns	Ns	*	Ns	Ns	Ns	Ns	Ns	Ns	Ns
	K × FM		Ns	Ns	Ns	Ns	Ns	*	Ns	Ns	Ns	Ns	Ns	Ns	Ns	Ns	Ns	Ns
	K × CE		Ns	Ns	Ns	Ns	Ns	Ns	Ns	Ns	**	Ns	Ns	Ns	Ns	Ns	Ns	Ns

Different lowercase letters in the same column indicate significant differences between different groups (*P* < 0.05) for “N1” or “Xu28”. Asterisks denote significant differences after AMF inoculation (*t*-test; *P < 0.05; **P < 0.01; ***P < 0.001). Ns denotes not significant using Tukey’s test.

### Correlation analysis with root morphological index and the K uptake of sweet potato

**FIGURE 8:**
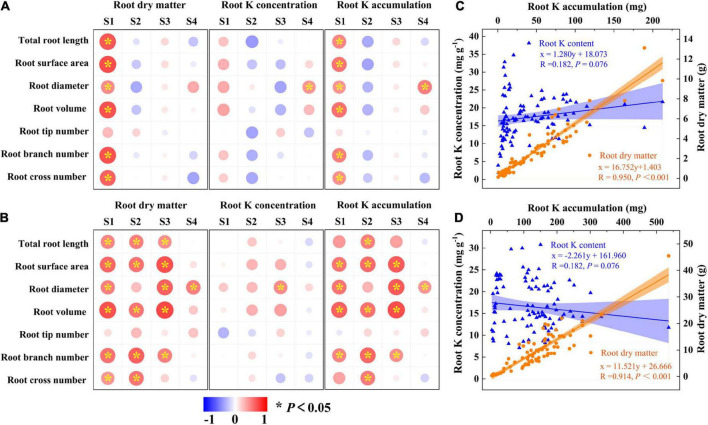
Pearson’s correlation coefficients between root morphological index, root dry matter, root K concentration, and root K accumulation of N1 **(A)** and Xu28 **(B)**. Relationship between root K concentration or root dry matter and root K accumulation of N1 **(C)** and Xu28 **(D)**. Asterisks denote significant differences (*P < 0.05). The value of R and *P* given in each figure to exhibit the significant level (*P* < 0.05, significant; *P* ≥ 0.05, nonsignificant).

The above results showed that K and CE displayed a synergistic effect on root development and K acquisition especially for “Xu28”. Pearson’s correlations revealed that root morphological index showed positive correlations with root DM and root K accumulation of “N1” only at seeding stage (Figure 8A), while root morphological index showed positive correlations with root DM and K accumulation of “Xu28” from seeding stage to harvesting stage (Figure 8B). No significant positive correlations were observed between root morphological index and K concentration of “N1” or “Xu28” from seeding to harvesting stage, except that root diameter showed significant positive correlation relationships with root K concentration at harvesting stage of “N1” and at maturing stage of “Xu28” (Figures 8A, B). In addition, root K accumulation showed significant positive correlation relationship with root DM rather than root K concentration for both “N1” and “Xu28” (Figures 8C, D). Therefore, the increased root DM was explained by the optimized morphology of sweet potato for N1 (particularly at seeding stage) and Xu28 (from seeding to harvesting stage), and the increased root K accumulation was attributed to the increased root DM rather than root K concentration of sweet potato.

## Discussion

Sweet potato has higher requirements for K for optimum production (Tang et al., 2015), and is easily colonized by AMF (O’Keefe and Sylvia, 1993; Minemba et al., 2019). Meanwhile, optimal K nutrient management strategy for sustainable and environmentally protective crop production was using KIUE genotypes in combination with appropriate soil fertilization (Romheld and Kirkby, 2010). It is acknowledged that root architecture plays a fundamental role for water and nutrients uptake from soil and in turn, yield production (Alhadidi et al., 2021; Caruso et al., 2021). Optimizing root morphology of sweet potato are effective methods guaranteeing yield production of sweet potato. Previously, K application and AMF inoculation have been proven to exert great effects on the root morphology regulation and nutrient acquisition alternation of sweet potato (Alhadidi et al., 2021; Gao et al., 2021), and the interaction exists between K and KIUE in sweet potato (Wang et al., 2017). In the present study, K application differentially regulated the root architecture of “N1” and “Xu28”, and K application showed greater effect on root architecture optimization of high-KIUE genotype “Xu28” (Figure 3). The defined impact of AMF on plant growth is not stable considering the interaction with AMF and the environmental conditions. It is worth noting that either FM or CE inoculation had little effect on the root morphology of sweet potato and tended to have negative effect without K application, while FM and CE inoculation significantly optimized root morphology under the condition with K application (Figure 4), indicating that AMF inoculation-improved root morphology of sweet potato highly relied on K application. Similarly, both K application and AMF inoculation promote root growth and K accumulation of wolfberry, and a synergistic interaction existed between K and AMF (Wang, 2020). Likewise, plant K concentration modified the effects of AMF on root hydraulic properties in maize roots (El-Mesbahi et al., 2012). Therefore, we deduced K application and AMF inoculation exhibited a considerable synergistic effect on the root morphology of sweet potato both low- and high-KIUE genotypes.

Genetic and environmental factors collectively determine plant growth and yield (Wang et al., 2022b). Similarly, sweet sorghum genotypes were selectively associated with AMF inoculations species (FM, *Claroideoglomus claroideum* and CE) for plant root parameters and nutrient uptake (Ortas and Bilgili, 2022). The interaction amongst AMF species may differ between sweet potato varieties (Yooyongwech et al., 2016). On the one hand, plant genotypes were considered in this study. Root morphological indexes (root DM, total root length, root surface area, root volume, root diameter, root branch number, and root tip number) should be analyzed when checking root growth state (Alhadidi et al., 2021; Caruso et al., 2021). As shown in the recent study, *Glomus intraradices* induced different root architecture models (length, surface area, average diameter, and biomass) in relation to the cultivars of *Ficus carica*, suggesting diverse root strategies for exploiting the soil resources (Caruso et al., 2021). In this study, FM and CE inoculation increased root tip number of “N1” in the swelling stage and optimized multiple indexes of root morphology of “Xu28” during the whole growth stage under the condition with K application (Figure 4), indicating that AMF differentially regulated root morphology of low-KIUE and high-KIUE sweet potato. On the other hand, AMF species were considered in this study. Three different AMF species (i.e., *C. claroideum*, *Rhizoglomus irregulare*, and FM) varied in their effects on plant biomass and nutrient acquisition of different host plant (Köhl and van der Heijden, 2016). In this study, FM and CE were more favorable to optimize root morphology of “N1” and “Xu28”, respectively (Figure 4), CE rather than FM had the best optimization effect on the root morphology of “Xu28” under the condition with K application (Figure 4 and Supplementary Figure 2), indicating that AMF species greatly affected root morphology of sweet potato. Therefore, we speculated that both sweet potato genotypes and AMF species affected the outcome of benefits that AMF conferred to sweet potato to a great extent.

**FIGURE 9:**
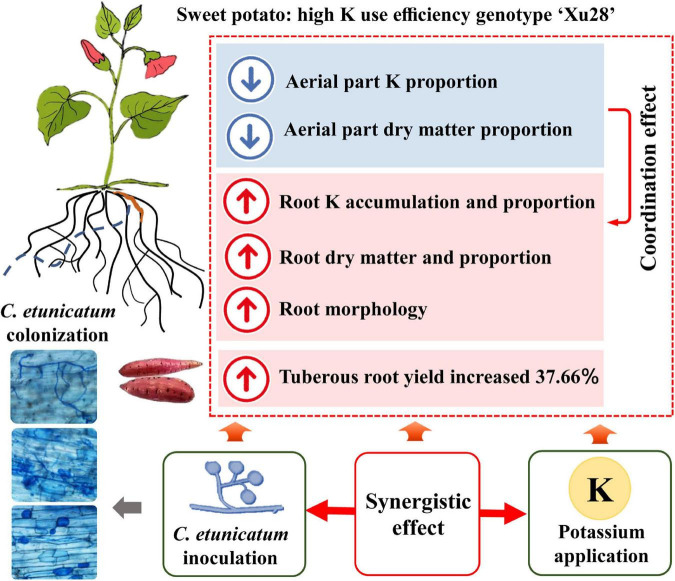
Conceptual diagram depicting the synergistic effect of K and arbuscular mycorrhizal fungi (AMF) on root development and K acquisition of high K use efficiency genotype “Xu28”.

Consistent with the optimized root morphology of “Xu28” (Figure 4 and Supplementary Figure 2), CE inoculation significantly promoted root DM accumulation of “Xu28” in the swelling and harvesting stages (Figure 5), and coordinated of aerial part and root growth of “Xu28”, reduced the DM to leaf and petiole, be beneficial to DM allocation to the root under conditions of K supply (Figure 6). Likewise, CE inoculation could limit K allocation to the aboveground and promote root K accumulation of “Xu28” under the condition with K application (Tables 1, 2). The above results lead to the interesting finding that K and CE displayed a synergistic effect on root development and K acquisition of high-KIUE genotype “Xu28”. Moreover, CE inoculation, and K application and CE inoculation interaction significantly increased tuberous root yield of “Xu28” rather than “N1” (Figure 2), indicating that K application and CE inoculation exhibited a synergistic effect on tuberous root yield of “Xu28”. Therefore, CE inoculation under the condition of K application is an effective strategy to improve the yield production of high-KIUE genotype “Xu28”. Thus, we proposed that K application and CE inoculation synergistically optimized root morphology, improved root DM and root K accumulation, coordinated the aerial part and root growth and K allocation, and increased tuberous root production of high-KIUE genotype “Xu28” (Figure 9). In this study, −FM or −CE was set as the control of +FM or +CE, respectively, to replenish the interference from other factors such as mineral and non-mycorrhizal microbial components in the living inoculums. It’s worth noting that there are some differences between −FM and −CE in the same genotype and same K level (Table 1). For −FM or −CE, AMF inoculum (70 g) of FM or CE was sterilized and filtered through a 25-μm membrane, respectively. And, to replenish the mineral and non-mycorrhizal microbial components in the living inoculums, the obtained microbial filtrate of unsterilized FM or CE inoculum was inoculated into the pot of −FM or −CE, respectively. Thus, the −FM treatment and −CE treatment were not the same treatment, and some metabolite may also mediate these differences. Considering more complex and uncertain environmental factor existing in field, and environmental factor (soil types, soil acidity, carbon: nitrogen: phosphorus ratio in soil, climate, etc.) could affect the functional effect of AMF in the agricultural field (Kalamulla et al., 2022), further studies will be conducted to investigate whether K application and CE inoculation improve root morphology and yield production in field experiments, which will contribute to more scientific application of AMF in sweet potato cultivation.

## Conclusion

This study showed that K application differentially regulated the root architecture of low-KIUE genotype “N1” and high-KIUE genotype “Xu28”, FM and CE inoculation were more inclined to optimize root morphology of “N1” and “Xu28”, respectively. AMF inoculation-improved root morphology of sweet potato highly relied on K application. Particularly, K application and CE inoculation displayed a synergistic effect on root development and K acquisition of high-KIUE genotype “Xu28”. This study provides a theoretical basis for more scientific application of AMF in sweet potato cultivation and helps to further clarify plant–nutrient–AMF interactions.

## Data availability statement

The original contributions presented in this study are included in this article/Supplementary material, further inquiries can be directed to the corresponding author.

## Author contributions

JY and JW: conceptualization. JY, LW, CX, HZ, and CS: methodology. JY, KS, and XZ: investigation. JY: writing—original draft preparation. JY, YZ, and JW: writing—review and editing and funding acquisition. GZ, YZ, and JW: supervision. All authors contributed to the article and approved the submitted version.
