# Supplementary material for: Interactive impact of potassium and arbuscular mycorrhizal fungi on the root morphology and nutrient uptake of sweet potato (Ipomoea batatas L.)
Source: Front Microbiol. 2023 Jan 9;13:1075957. doi: 10.3389/fmicb.2022.1075957 (PMC9869065; doi:10.3389/fmicb.2022.1075957)
Supplement: Supplementary file 1 [file Data_Sheet_1.docx]

Supplementary Material

Interactive Impact of Potassium and Arbuscular Mycorrhizal Fungi on Root Morphology and Nutrient Uptake of sweet potato (*Ipomoea batatas* L.)


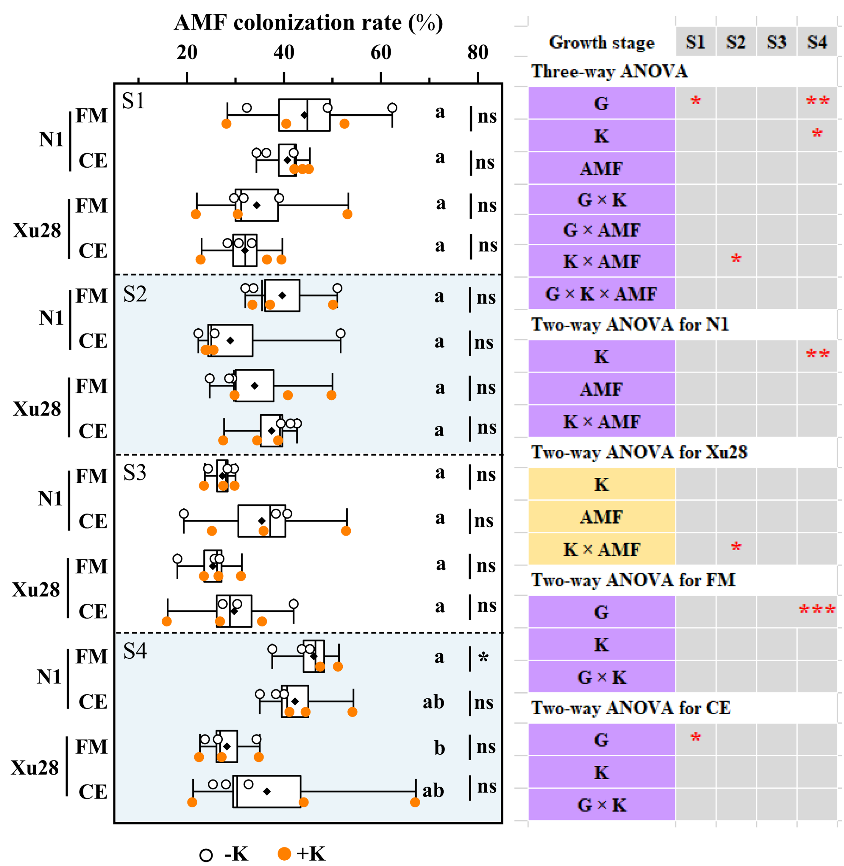


# Supplementary Figure S1. The effect of K application on AMF colonization rate in root of sweet potato. For each growth stage, different lowercase letters indicate significant differences between different group (*P* < 0.05). For different groups, asterisks denote significant differences after K application (*t*-test; **P* < 0.05; ***P* < 0.01), while “ns” indicates nonsignificant difference. ‘N1’ and ‘Xu28’ indicate “sweet potato ‘N1’” and “sweet potato ‘Xu28’”, respectively. “-K” and “+K” indicate “without potassium fertilizer” and “with potassium fertilizer”, respectively. S1, S2, S3 and S3 indicate seedling stage, swelling stage, maturing stage, and harvesting stage, respectively. “FM” and “CE” indicate “*Funneliformis mosseae*” and “*Claroideoglomus etunicatum*”, respectively.


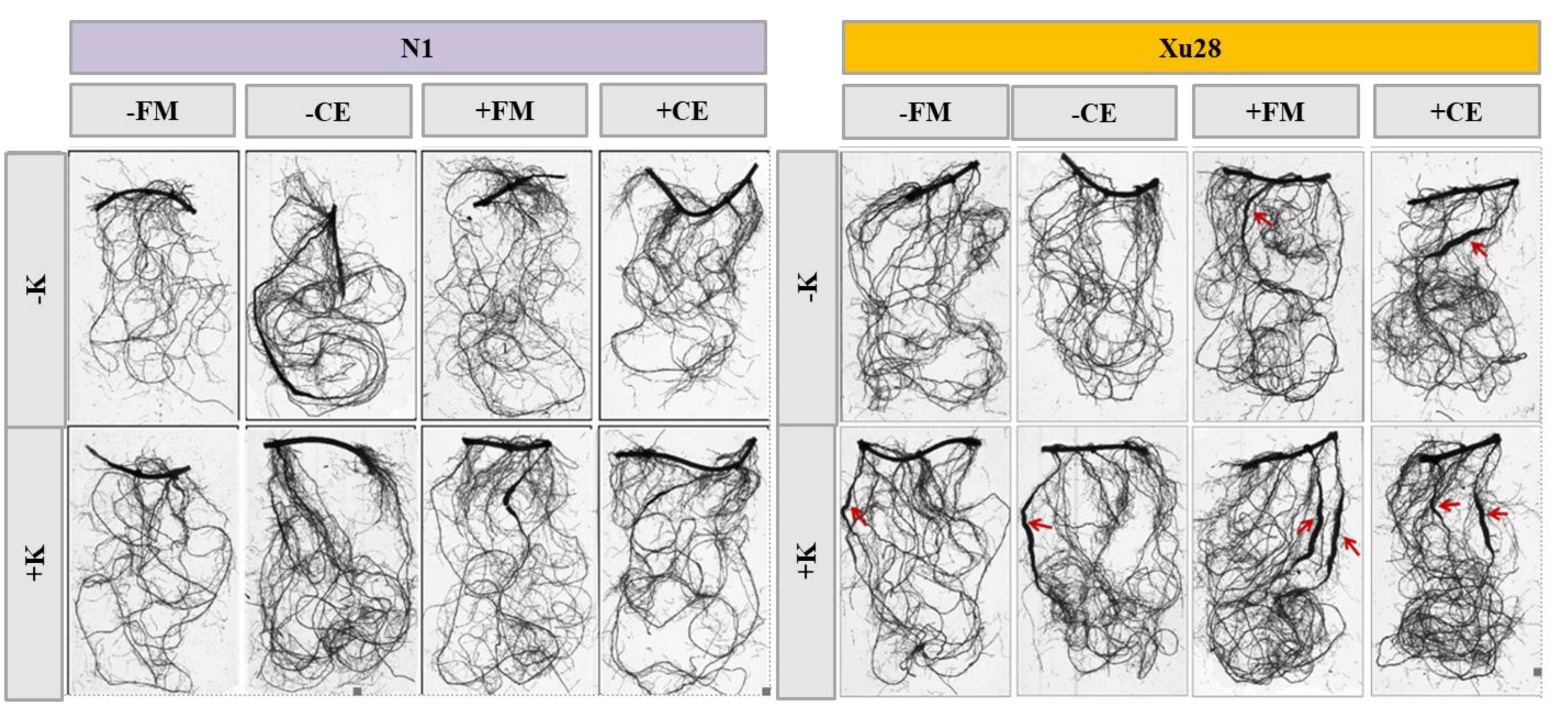


# Supplementary Figure S2. The effects of K application and AMF inoculation on root morphology of sweet potato at seeding stage. ‘N1’ and ‘Xu28’ indicate “sweet potato ‘N1’” and “sweet potato ‘Xu28’”, respectively. “-K” and “+K” indicate “without potassium fertilizer” and “with potassium fertilizer”, respectively. “FM” and “CE” indicate “*Funneliformis mosseae*” and “*Claroideoglomus etunicatum*”, respectively. Red arrows indicate swelling roots.


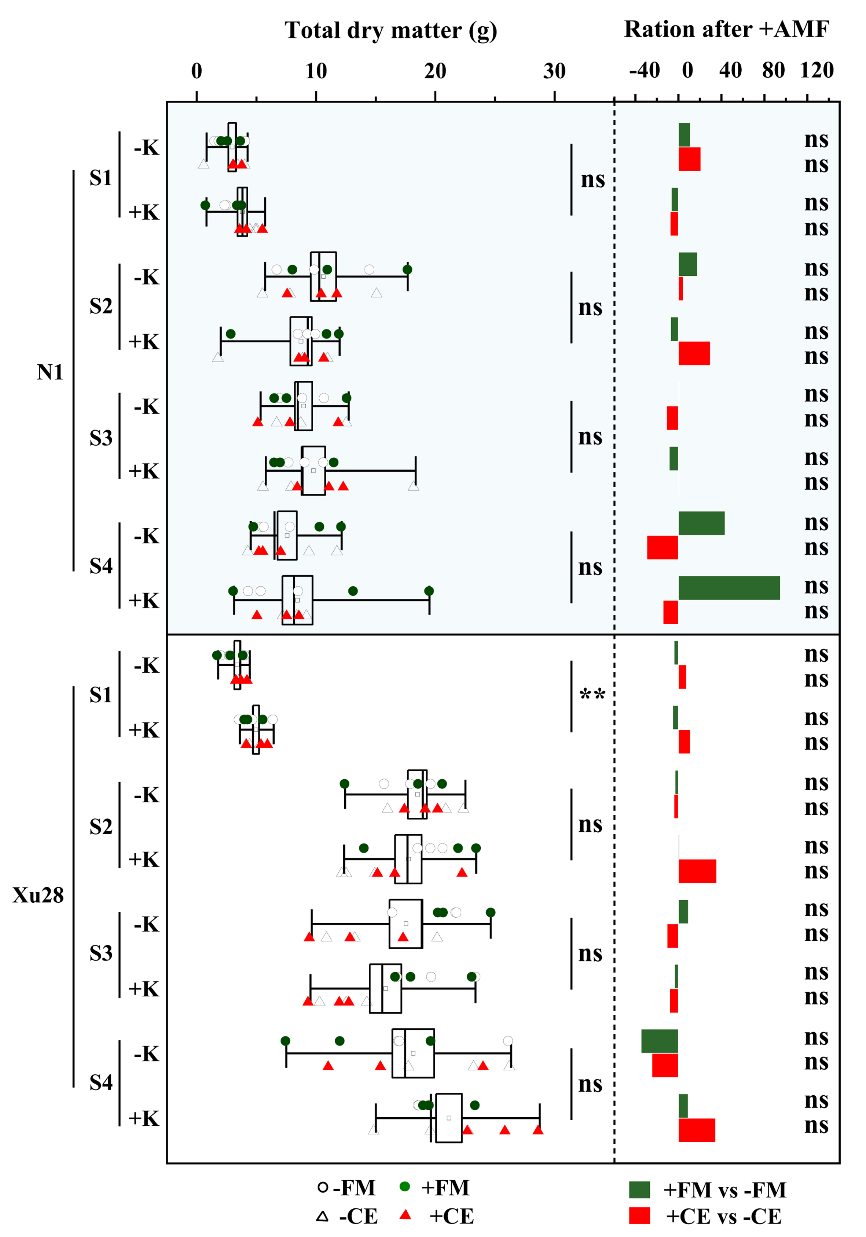


# Supplementary Figure S3. The effects of K application and AMF inoculation on total dry matter of sweet potato. For each growth stage, different lowercase letters indicate significant differences between different group (*P <* 0.05). For different groups, asterisks denote significant differences after AMF inoculation (*t*-test; **P* < 0.05; ***P* < 0.01), while “ns” indicates nonsignificant difference. ‘N1’ and ‘Xu28’ indicate “sweet potato ‘N1’” and “sweet potato ‘Xu28’”, respectively. “-K” and “+K” indicate “without potassium fertilizer” and “with potassium fertilizer”, respectively. S1, S2, S3 and S3 indicate seedling stage, swelling stage, maturing stage, and harvesting stage, respectively. “-FM”, “+FM”, “-CE”, “+CE” indicate “inoculation without FM”, “inoculation with FM”, “inoculation without CE” and “inoculation with CE”, respectively. “FM” and “CE” indicate “*Funneliformis mosseae*” and “*Claroideoglomus etunicatum*”, respectively. AMF indicate arbuscular mycorrhizal fungi.


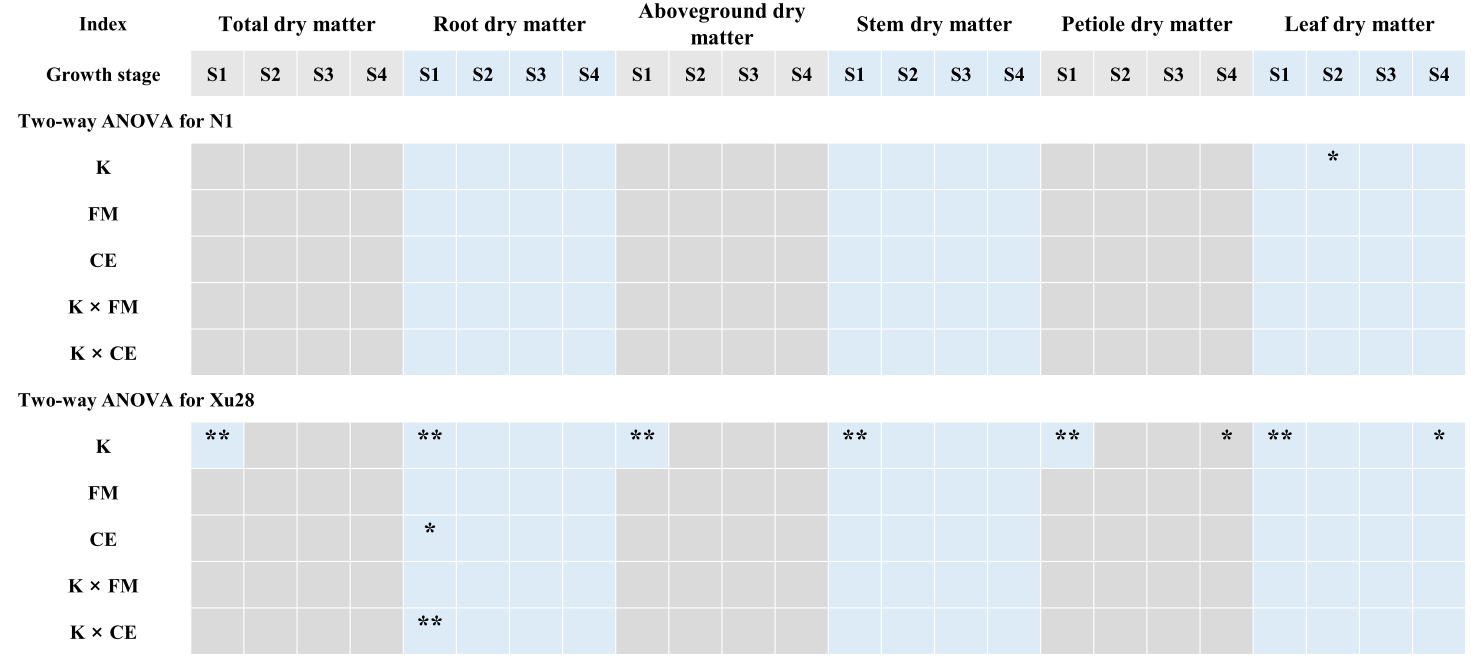


# Supplementary Figure S4. Two-way ANOVA analysis of variance for the effects potassium (K) and *F. mosseae* (FM) or *C. etunicatum* (CE) inoculation on dry matter of different organs of sweet potato. Asterisks denote significant differences (*t*-test; **P* < 0.05; ***P* < 0.01).


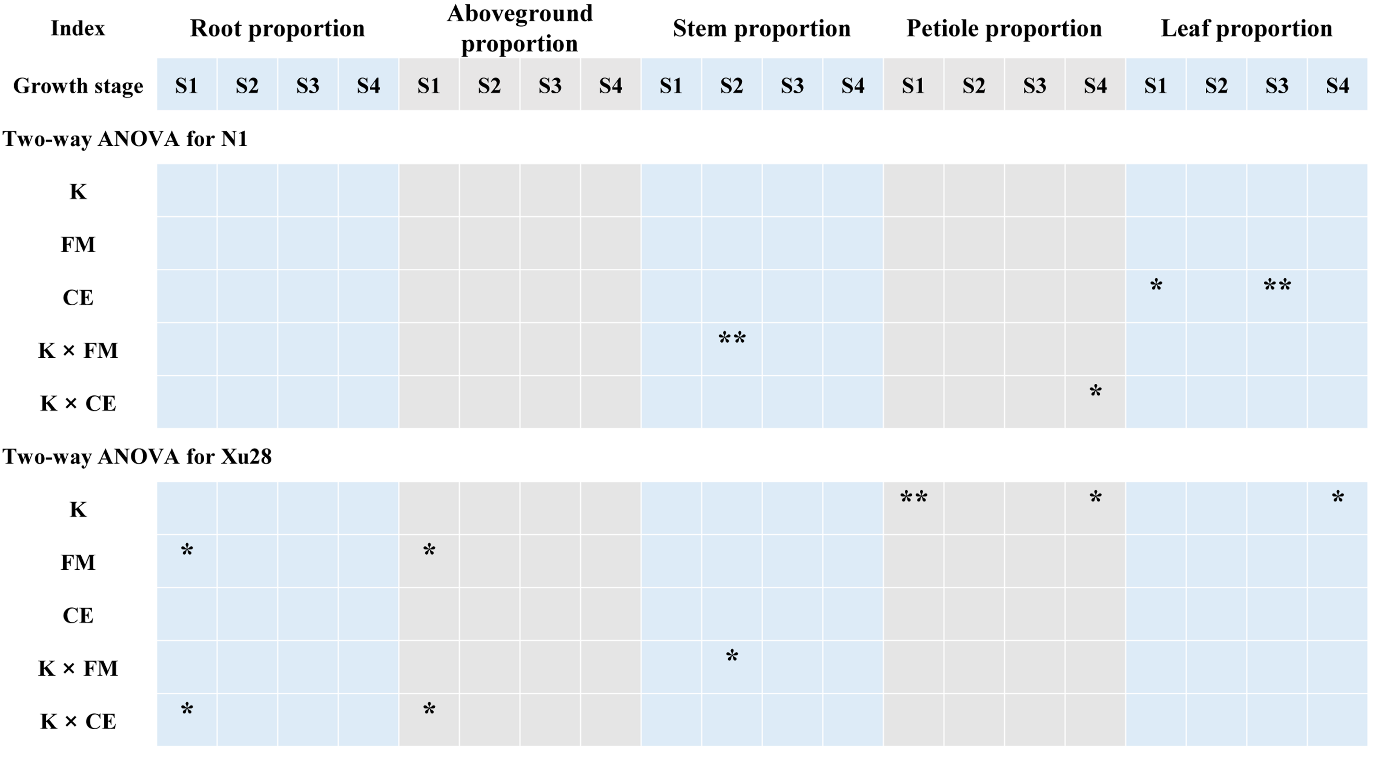


# Supplementary Figure S5. Two-way ANOVA analysis of variance for the effects potassium (K) and *F. mosseae* (FM) or *C. etunicatum* (CE) inoculation on dry matter proportion of different organs of sweet potato. Asterisks denote significant differences (*t*-test; **P* < 0.05; ***P* < 0.01).
